# Efectividad de mepolizumab y benralizumab en una cohorte de pacientes con asma grave eosinofílico en vida real

**DOI:** 10.1016/j.opresp.2021.100103

**Published:** 2021-04-28

**Authors:** María José Espinosa de los Monteros Garde, Víctor Romero Sanz, Cristina Blázquez Romero

**Affiliations:** a Servicio de Neumología, Hospital Virgen de la Salud, Toledo, España; b Servicio de Farmacia, Hospital Virgen de la Salud, Toledo, España

**Keywords:** Asma, Mepolizumab, Benralizumab, Asma grave eosinofílico, Vida real, Asthma, Mepolizumab, Benralizumab, Severe eosinophilic asthma, Real life

## Abstract

**Introducción:**

El mepolizumab es un anticuerpo monoclonal que bloquea la interleuquina 5 circulante (IL5). El benralizumab se une a la subunidad alfa del receptor de la IL 5 induciendo la eliminación directa de eosinófilos y basófilos. El propósito fue evaluar su efectividad en nuestra práctica clínica habitual.

**Métodos:**

Estudio observacional retrospectivo de una cohorte de pacientes asmáticos graves eosinofílicos tratados con mepolizumab y benralizumab durante al menos 6 meses. El seguimiento se ha realizado en la unidad de asma.

**Resultados:**

Se analizaron 25 pacientes en tratamiento con mepolizumab y 13 con benralizumab alcanzando un tiempo de seguimiento de 12 meses en el 73,7% de los mismos (10 en el caso de benralizumab y 18 para mepolizumab). Tras el tratamiento biológico se observa una disminución del 71% en el número de ingresos (p = 0,0027) y del 66% (p = 0,0013) en las visitas a urgencias. Se contabilizaron 31 ciclos de corticoides vs. 15 tras el tratamiento biológico suponiendo un descenso de un 52% (p = 0,0069). El control del asma medido por cuestionario de control (ACT) fue de 10,8 puntos (SD: 4,73) vs. 20,3 puntos (SD: 4,77), (p = 0,001) y el % FEV_1_ fue del 70,6% (SD: 21.5) vs. 76,2% (SD: 27,2), (p = 0,0437).

**Conclusiones:**

Se confirma la efectividad de los fármacos anti-IL5 en vida real mostrando mejoras en exacerbaciones, control y función pulmonar.

## Abstract (en)

**Introduction:**

Mepolizumab is a monoclonal antibody that inhibits circulating interleukin 5 (IL-5). Benralizumab binds to the alpha subunit of the IL-5 receptor, inducing the direct elimination of eosinophils and basophils. The aim of this study was to evaluate their effectiveness in our routine clinical practice.

**Methods:**

A retrospective observational study of a cohort of patients with severe eosinophilic asthma treated with mepolizumab and benralizumab for at least 6 months, and followed up in the asthma unit.

**Results:**

We analyzed 25 patients who received mepolizumab and 13 who received benralizumab, 73.7% of whom were followed up for 12 months (10 months for benralizumab and 18 for mepolizumab). After biological treatment, there was a 71% reduction in the number of admissions (p = .0027) and a 66% reduction (p = .0013) in emergency visits. A reduction in corticosteroid use was recorded: 31 cycles of corticosteroids compared to 15 after biological treatment, representing a decrease of 52% (p = .0069). Asthma control measured by the Asthma Control Test was 10.8 (SD: 4.73) vs. 20.3 (SD: 4.77), (p = .001) and FEV_1_% was 70.6% (SD: 21.5) vs. 76.2% (SD: 27.2), (p = .0437).

**Conclusions:**

The effectiveness of anti-IL-5 drugs in real life was confirmed, with improvements in exacerbations, asthma control and lung function.

## Introducción

El asma grave es la que requiere múltiples fármacos y en altas dosis para mantener el control (escalones 5 y 6 de la Guía Española para el Manejo del Asma [GEMA]^1^y 5 de la Global Initiative for Asthma [GINA]^2^), o la que permanece mal controlada a pesar de estos escalones de tratamiento. El asma eosinofílico supone algo más del 25% de los asmas graves y se caracteriza por la presencia de eosinófilos en las biopsias bronquiales y en el esputo, a pesar de un tratamiento con dosis altas de glucocorticoides. Pueden cursar con rinosinusitis crónica y pólipos nasales y un subgrupo desarrolla enfermedad respiratoria exacerbada por aspirina (EREA). Una elevada producción de interleuquina 5 (IL-5) puede explicar la inflamación eosinofílica en ausencia del clásico mecanismo T2 mediado por la alergia^3^, ^4^. En los últimos 20 años se han ido diferenciando los fenotipos de asma grave no controlado^5^. Disponemos en la actualidad de tres medicamentos cuyo mecanismo de acción es el bloqueo de IL-5, mepolizumab^6^, benralizumab^7^ y reslizumab^8^. Para el caso de mepolizumab los estudios a 4 años demuestran un perfil favorable de seguridad y un efecto estable y duradero^9^, ^10^. En el caso del benralizumab estudios de seguimiento a 2 años validan los resultados de eficacia y seguridad^11^. Son escasos los estudios en vida real que evalúen el impacto de los fármacos biológicos en el tratamiento del asma. Dichos trabajos son claves a la hora de objetivar el efecto del tratamiento en condiciones de práctica clínica habitual. El propósito del estudio fue evaluar las variables de efectividad y función pulmonar entre el periodo prebiológico (al menos 6 meses antes del inicio de la pauta del tratamiento) y el posbiológico.

## Material y métodos

Se lleva a cabo un estudio observacional retrospectivo en la Unidad de Asma del Hospital Virgen de la Salud de Toledo en el que se incluyeron todos los pacientes asmáticos graves eosinofílicos tratados con biológicos (mepolizumab o benralizumab) durante al menos 6 meses. Todos fueron diagnosticados de asma mediante una historia clínica compatible junto a una espirometría con test de broncodilatador (reversibilidad del FEV1 ≥12%, test de metacolina positiva o variabilidad del FEV1 ≥20%). Nos aseguramos, antes del inicio del biológico, tanto de la correcta adherencia a los fármacos como de la adecuada técnica de inhalación, implementamos un plan educativo, identificamos posibles desencadenantes y/o agravantes de las exacerbaciones, ajustamos el tratamiento y evaluamos las comorbilidades. De acuerdo con los criterios de asma grave, estos pacientes recibían tratamiento de mantenimiento con una combinación de GCI/LABA a dosis elevadas, correspondiente al escalón 5 de la GINA o 5-6 según la GEMA con al menos unos de los siguientes fármacos: tiotropio o antileucotrieno. El inicio de benralizumab se estableció por los siguientes criterios: ≥18 años en asma refractario severo^12^; estadio 5 de GINA^2^; ≥2 exacerbaciones en el año previo con uso de corticoides sistémicos (OCS) a pesar de recibir un correcto tratamiento correspondiente al grado de severidad o corticodependencia; presencia de eosinofilia ≥300 cells/μ en sangre en el año previo o de ≥ 150 cells/μL en caso de corticodependencia. El inicio de mepolizumab se estableció por los siguientes criterios: ≥ 6 años con asma refractario severo^12^; estadio 5 de GINA^2^; ≥2 exacerbaciones en el año previo o uso de OCS a pesar de recibir un correcto tratamiento correspondiente al grado de severidad o corticodependencia; presencia de eosinofilia ≥ 300 cells/μ en sangre en el año previo último o ≥ 150/ μ en el momento del tratamiento, pero con controles históricos de eosinofilia. Todos los pacientes que fueron tratados con benralizumab o mepolizumab en los que pudiéramos hacer análisis comparativo pre- y posbiológico durante al menos 6 meses fueron incluidos en el estudio. Aquellos que previamente habían sido tratados con otro biológico pero que tuvieron respuesta fallida a juicio del clínico fueron incluidos también. Los siguientes criterios indicaban una falta de respuesta al biológico: continuidad de corticoides orales a pesar del tratamiento biológico tras 12 meses o < 50% en la reducción de exacerbaciones después de al menos 12 meses con el biológico. El registro respeta los principios éticos básicos de no maleficencia, justicia, autonomía y beneficencia y sigue las normas éticas de la Declaración de Helsinki, promulgada por la Asociación Médica Mundial en junio de 1964 y modificaciones posteriores. Asimismo, respeta en todo momento la intimidad, privacidad, confidencialidad y la protección de datos de los pacientes. La protección de datos están regulados en función del Reglamento (UE) 2016/679 del Parlamento Europeo y del Consejo de 27 de abril de 2016 de Protección de Datos y la Ley Orgánica 3/2018, de 5 de diciembre, de Protección de Datos Personales y garantía de los derechos digitales y la Ley 41/2002, de 14 de noviembre, básica reguladora de la autonomía del paciente. Se obtuvo el consentimiento informado de los asmáticos participantes. El proyecto de registro de biológicos fue aprobado por el Comité ético del Hospital Virgen de la Salud de Toledo. Se recogieron en el momento de inicio del biológico datos demográficos, presencia de atopia (rinoconjuntivitis, dermatitis atópica, alergia a alimentos), tratamiento previo con otro fármaco biológico, corticodependencia (entendida como el uso diario de corticoides sistémicos durante al menos 6 meses), presencia de EREA o rinitis, control del asma medido por el cuestionario de control (ACT) e índice de masa corporal, porcentaje del volumen espirado en el primer segundo (FEV_1_) y eosinófilos en sangre. Las siguientes variables fueron recogidas 6 meses prebiológico vs. 6 meses posbiológico y en el caso de 28 pacientes 12 meses prebiológico vs. 12 meses posbiológico: eran variables de efectividad como las exacerbaciones definidas por: número de ingresos hospitalarios (media y desviación estándar), admisiones al servicio de urgencias (media y desviación estándar) y necesidad de número de ciclos de corticoides (≥3 días consecutivos).

## Análisis estadístico

Para la extracción de los datos de los pacientes se utilizó la historia clínica electrónica mediante el sistema de información hospitalaria MAMBRINO XXI® y se recogieron las variables en una base de datos de Excel®. El tratamiento estadístico de los datos se realizó mediante el programa STATA/MP® 16.0. El supuesto de normalidad se ha evaluado mediante métodos gráficos y la prueba de Shapiro-Wilk. Las comparaciones entre el estado basal y el de seguimiento se realizó con la prueba no paramétrica T de Wilcoxon. Las variables cualitativas se presentan con frecuencias y proporciones y las variables cuantitativas se presentan como medidas de tendencia central (media) y de dispersión (desviación estándar [SD]). Se consideró un valor de p < 0,05 para la significación estadística.

## Resultados

*Características clínicas:* se revisaron un total de 38 pacientes con diagnóstico de asma grave eosinofílico que recibieron tratamiento con un fármaco biológico durante al menos 6 meses. El 65,8% (25 pacientes) fueron tratados con mepolizumab, 76% durante al menos 12 meses y el 34,2% (13 pacientes) con benralizumab, 69,2% durante al menos 12 meses. El 26,3% (10 pacientes), habían recibido previamente otro fármaco biológico, 8 en el grupo de benralizumab, siendo el fármaco biológico previo omalizumab en 2 pacientes y mepolizumab en 6 pacientes. Omalizumab fue el fármaco biológico previo en 2 pacientes del grupo de mepolizumab. El 10,5% (4 pacientes) eran corticodependientes, todos ellos tratados con mepolizumab. Se suspendieron 3 mepolizumab y 2 benralizumab por inadecuada respuesta, no obstante, entraron en el análisis cumplimentando al menos 6 meses de tratamiento.

Las características basales de los pacientes son presentadas en la tabla 1. *Parámetros analizados:* se compararon las variables de efectividad y función pulmonar entre el periodo prebiológico (al menos 6 meses antes del inicio de la pauta del tratamiento) y el posbiológico (tabla 2). La media de exacerbaciones que requirieron asistencia sanitaria en relación exclusivamente a ingresos y/o visitas a urgencias fue de 2,18 (SD: 2,62) frente a 0,71 (SD:1,52) lo que supone una disminución de 1,47 exacerbaciones por paciente (p = 0,0005). En el caso de mepolizumab pasamos de una media de 2,28 exacerbaciones (SD: 2,55) a 0,96 (SD:1,79) (p = 00074), mientras que para el grupo de benralizumab pasamos de 2 exacerbaciones (SD: 2,8) a 0,23 (SD: 0,59) (p = 0,0313). El análisis estadístico por subgrupos queda reflejado en las Tabla 3, Tabla 4. Además, en cuanto a los datos del cuestionario ACT globalmente se consiguió aumentar el valor en 9,5 puntos. En el grupo de mepolizumab se pasó de 11,4 puntos (SD: 4,89 puntos) a 20,9 puntos (SD: 4,54 puntos) (p = 0,0006), en el grupo de benralizumab se pasó de una media de 10 puntos (SD: 4,73 puntos) a 19,3 puntos (SD: 5,14 puntos) (p = 0,002). En relación a la eosinofilia, el 15,3% de los pacientes con benralizumab tenían eosinofilia < 300 cells/μL, siendo del 32% para mepolizumab. Para el conjunto de los 38 pacientes, la media de eosinófilos antes del tratamiento con el fármaco biológico fue de 420,1 cells/μL (SD: 302,8 cells/μL) frente a 93,4 cells/μL (SD: 123,8 cells/μL) tras el tratamiento (p = 0,0001). Analizando los datos por subgrupos, para benralizumab la media de eosinófilos pretratamiento fue de 568,3 cells/μL (SD: 412,5 cells/μL), siendo de 18,7 cells/μL (SD: 46,7 cells/μL) tras el tratamiento (p = 0,0156). En el grupo de mepolizumab, la media de eosinófilos prebiológico fue de 362,4 cells/μL (SD: 238,4 cells/μL), siendo de 126,1 cells/μL (SD: 133,7 cells/μL) tras el tratamiento (p = 0,0076). Finalmente, encontramos una mejoría significativa del % FEV_1_ tras al menos 6 meses de tratamiento pasando de una media de 70,6% (SD: 21,51%) a 76,2% (SD: 27,29%), lo que supone un aumento porcentual del % FEV_1_ de 5,6% (p = 0,0437), sin embargo por subgrupos la mejora no fue significativa ya que para el grupo de mepolizumab se pasó de un % FEV_1_ medio de 72,1% (SD: 24,35%) a 77,9% (SD: 32,07%) (p = 0,1393) y para benralizumab se pasó de una media de 67,3% (SD: 13,84%) a 72,3% (SD: 11,88%) (p = 0,1914). De los 4 pacientes corticodependientes, 3 de ellos tuvieron una respuesta total al tratamiento con un biológico permitiendo la retirada de los corticoides (tabla 5). Ningún otro paciente varió su tratamiento farmacológico habitual tras el biológico.

**Tabla 1:** Características basales de los pacientes tratados con anti-IL 5

N = 38 pacientes	Anti-IL 5 (mepolizumab + benralizumab)	Mepolizumab (N = 25)	Benralizumab (N = 13)
Edad (años), media (SD) (rango)	64,2 (13,5) (23-58)	65	62
Mujeres, n (%)	23 (60,5)	13	10
Tratamiento biológico previo, n (%)	10 (26,3)	2	8
Atopia, n (%)	10 (8)	3	7
Corticodependiente, n (%)	4 (10,5)	4	0
Síndrome EREA/rinitis, n (%)	9 (23,7)	6	3
ACT, media (SD)	10,8 (4,7)	11,4 (4,9)	10 (4,73)

ACT: Cuestionario de control del asma; IL5: interleuquina 5.

**Tabla 2:** Exacerbaciones antes y después del tratamiento biológico

Variables	Período prebiológico media (SD)	Período posbiológico media (SD)	Diferencia (%)	P*
Número visitas a urgencias	1,44 (1,8)	0,50 (1,2)	-66%	0,0013
Número ingresos	0,74 (1,1)	8 (0,21)	-71%	0,0027
Ciclos de esteroides	0,8 (0,9)	0,4 (0,7)	-52%	0,0069

* Comparación de los datos antes y después del tratamiento biológico.

**Tabla 3:** Exacerbaciones antes y después del tratamiento con mepolizumab

Variables mepolizumab	Período prebiológico media (SD)	Período posbiológico media (SD)	Diferencia (%)	P*
Número ingresos	0,80 (1,1)	0,32 (0,6)	-60%	0,04
Número urgencias	1,48 (1,6)	0,64 (1,4)	-57%	0,015
Ciclos de esteroides	0,96 (1,1)	0,44 (0,8)	-54%	0,02

* Comparación de los datos antes y después del tratamiento biológico.

**Tabla 4:** Exacerbaciones antes y después del tratamiento con benralizumab

Variables benralizumab	Período prebiológico media (SD)	Período posbiológico media (SD)	Diferencia (%)	P*
Número ingresos	0,62 (0,9)	0	-100%	0,06
Número urgencias	1,38 (2,1)	0,23 (2,1)	-83%	0,07
Ciclos de esteroides	0,54 (0,5)	0,31 (0,5)	-43%	0,37

* Comparación de los datos antes y después del tratamiento biológico.

**Tabla 5:** Características clínicas y funcionales comparativas entre periodo posbiológico

Variables	Período prebiológico media (SD)	Período posbiológico media (SD)	Diferencia	P*
ACT	10,8 (4,8)	20,3 (4,8)	9,5	0,001
FEV1%	70,6 (21,5)	76,2 (27,3)	5,6	0,0437
Eosinofilia	420,1 (302,8)	93,4 (123,8)	326,7	0,0001

ACT: Asma Control Test; FEV1: volumen espiratorio forzado en el primer segundo.

* Comparación de los datos antes y después del tratamiento biológico.

## Discusión

Los estudios en vida real pueden diferir de los datos obtenidos en los pivotales^13^. El presente estudio confirma que los fármacos biológicos anti-IL5, benralizumab y mepolizumab reducen las exacerbaciones, los ciclos de corticoides y mejoran la función pulmonar y el control en línea con los estudios pivotales fase III de benralizumab (SIROCCO y CALIMA)^14^, ^15^ y de mepolizumab (MENSA y DREAM)^16^, ^17^ y con otras series en vida real para benralizumab^18^, ^19^ y mepolizumab^20^. Observamos que en cuanto a la reducción de exacerbaciones nuestros resultados son superiores a los pivotales para benralizumab ya que mientras en SIROCCO se produjo una reducción a las 48 semanas del 51% vs. placebo (ratio, 0,49; 95% CI, 0,37-0,64) y en CALIMA a las 52 semanas del 28% vs. placebo (ratio, 0,72; 95% CI, 0,54-0,95), en nuestro caso alcanzamos reducciones del 95% (de 1,62 a 0,08) siendo del 100%(p = 0,06) para hospitalizaciones y del 83% (p = 0,07) para visitas a urgencias. El dato no alcanza la significación estadística, pero entendemos que se debe al escaso tamaño muestral. En relación a otros estudios en vida real^20^ se obtienen reducciones del número anual de exacerbaciones de 3,5 a 0, siendo en nuestra serie el descenso de 2,28 a 0,96. Para el caso de mepolizumab, debido al escaso número de pacientes corticodependientes no podemos extraer conclusiones de reducción como en el estudio pivotal SIRIUS^21^ pero sí reseñar que de los 4 pacientes, 3 de ellos abandonaron totalmente el uso regular de corticoides. Destacan los pocos pacientes corticodependientes en nuestra casuística sin encontrar clara explicación al respecto a excepción de la posible reticencia a su pauta de forma regular por los efectos adversos que ocasionan entendiendo su implementación en el último escalón terapéutico, a la dosis más baja eficaz y durante el mínimo tiempo posible. Además, hay una reducción significativa similar a los estudios pivotales en los que la tasa de exacerbaciones que requieren hospitalización o visita a urgencias fue del 61% en el grupo 100 mg sc (*P* = 0,02) (MENSA)^16^ o una reducción de exacerbaciones del 60% para 75 mg sc p < 0,0001(DREAM)^17^ y del 68% para MUSCA^22^ (P = 0,031). En nuestra serie la reducción de exacerbaciones fue del 58% (2,28 vs. 0,96) siendo del 60% (p = 0,04) para las hospitalizaciones y del 57% (p = 0,015) para las visitas a urgencias, similares resultados también a otros estudios en vida real como el REALITI-A^20^ con disminución de hospitalizaciones en un 55,8% y de visitas a urgencias del 55,7%. En relación a la función pulmonar para estudios de vida real como el de Miralles et al.^18^ se objetivaron mejoras de FEV1 del 47,5% al 60,5% al año o incrementos del 64,3% al 76,1% a los 6 meses como en el de Padilla et al.^19^, mostrando en nuestra serie un aumento más discreto ya que el % FEV1 varía del 67,3% al 72,3%, no alcanzando la significación estadística por subgrupos como consecuencia del pequeño tamaño muestral de los mismos aunque sí se alcanzó en el análisis global de ambos fármacos. Por otra parte, reseñar que hemos empleado el benralizumab con un nivel de eosinófilos ≥150- < 300 cells/μL en un 15,3% de los pacientes ya que estos procedían de pacientes con tratamientos biológicos previos fallidos en efectividad teniendo pólipos nasales, FVC < 65% y/o ≥ 3 exacerbaciones en el año previo. Benralizumab también muestra su eficacia en pacientes atópicos tal y como se demostró en un postanálisis de dos ensayos clínicos^23^. Finalmente, hemos alcanzado el control con 9,5 puntos de diferencia del ACT respecto al basal, sin embargo, en el análisis por subgrupos, aunque la diferencia fue significativa en ambos fármacos no se consiguió el control (ACT > 20) en el grupo de benralizumab, posiblemente debido al escaso número de pacientes. Nuestro estudio tiene limitaciones dado que no disponemos de grupo control y el tamaño muestral es muy pequeño cuando hacemos el análisis por subgrupos, sobre todo en el caso de benralizumab. Por ello, en algunas variables no es posible llegar a conclusiones estadísticamente significativas. Sin embargo, los resultados arrojan conclusiones clínicamente importantes, tratándose además de uno de los estudios que analizan el impacto de los fármacos biológicos en vida real con un mayor tiempo de seguimiento tras 12 meses de tratamiento (28 pacientes).

## Conclusiones

Nuestros datos muestran que los fármacos biológicos anti-IL5, mepolizumab y benralizumab empleados en pacientes con asma grave eosinofílica no controlada son efectivos en vida real mejorando la función pulmonar, la clínica de nuestros pacientes y reduciendo el número de visitas a urgencias, hospitalizaciones y ciclos de corticoides optimizando así el uso de recursos sanitarios, obteniendo resultados similares e incluso superiores a los estudios pivotales hasta la fecha realizados proporcionando un análisis en vida real con un tiempo prolongado de seguimiento demostrándose que son fármacos seguros y bien tolerados.

## Conflicto de intereses

Los autores declaran no tener ningún conflicto de intereses.
